# The effect of high-intensity versus photobiomodulation therapy (PBM) on the regeneration of the sciatic nerve following crush injury: an animal study

**DOI:** 10.1007/s10103-025-04334-w

**Published:** 2025-02-08

**Authors:** Kemal Atakan Bayburt, Nurettin Diker, Mehmet Serif Aydin, Dogan Dolanmaz

**Affiliations:** 1 Biruni University, Istanbul, Turkey; 2 Istanbul Medipol University, Istanbul, Turkey; 3 Bezmialem Vakif University, Istanbul, Turkey

**Keywords:** Sciatic nerve, Laser therapy, Laser biostimulation, Injuries, Nerve regeneration

## Abstract

The purpose of this research was to evaluate the therapeutics effects of photobiomodulation and high intensity laser therapy after a sciatic nerve crush injury. Following the crush injuries of sciatic nerve, 33 rats were randomly divided into three groups. The injured sciatic nerves of the rats in the control group were left to heal spontaneously, whereas HILT (120 J/session and 1064 nm) and photobiomodulation therapy (PBM) (2.4 J/session and 650 nm) were started immediately after surgery and performed once every 3 days (10 session in total) during the postoperative period. Electrophysiological evaluations were conducted before surgery and at the end of the healing period. The Sciatic Functional Index (SFI) was assessed before surgery and at the end of the healing period. The ratio of the inner axonal diameter to the total outer axonal diameter (g-ratio) and schwann cells per square micrometer were histomorphometrically evaluated. At the end of the 30-day healing period, significantly better SFI scores were noted in the HILT group compared with PBM (p=0.002) and control (p < 0.001) groups. HILT exhibited positive effects on latency and duration values when compared PBM (p=0.002, p=0.014) and control (p=0.003, p < 0.001) groups. The number of nerves with an optimum g-ratio was higher in the HILT group which indicates a better rate of myelination. Functional, histomorphometric, and electrophysiological investigations of the present study revealed that HILT seems to be a superior treatment modality for peripheral nerve regeneration.

## Introduction

Peripheral nerve injuries are a significant health issue, with an incidence of 13–23 cases per 100,000 individuals annually in developed countries. Such injuries may result in partial or complete loss of motor, sensory, and autonomic functions in the affected areas [1]. Particularly in dentistry, nerve injuries are commonly encountered during oral and maxillofacial surgeries. Contusions, trauma to the jaw and facial regions, dental implant procedures, local anesthesia injections, wisdom tooth extractions, pathologies in the maxillofacial area, or osteotomies performed to correct jaw deformities can lead to these injuries [2, 3]. These conditions cause functional loss and reduced quality of life in patients [3].

The most commonly used animal model for evaluating peripheral nerve injury and regeneration is the rat. The peripheral nervous system of rats shares similarities with that of humans, and their long, thick, and easily dissectible nerve structures make them a preferred model for research. The sciatic nerve, in particular, allows simultaneous evaluation of both motor and sensory functions. Its polyfascicular structure and the inclusion of different types of axons enable comprehensive examination in nerve regeneration studies [4–7]. In rat models, nerve injury is often induced using compression or transection methods. The compression model disrupts axons without compromising the integrity of the nerve trunk (epineurium integrity), creating an optimal regeneration environment in the distal segment. However, factors such as neuroma formation and scar tissue may hinder axonal regeneration, limiting functional recovery [8].

Various methods have been developed over time to promote nerve regeneration. Surgical techniques include nerve grafts and conduits, while non-invasive approaches include ultrasound, shock wave therapy, light-emitting diodes, and low-level laser therapy (LLLT). Studies have demonstrated that LLLT positively impacts sciatic nerve injuries in rats, increasing myelin sheath thickness [9], axon diameter [10], Schwann cell proliferation [11], and the expression of neurotrophic growth factors [12].

High-intensity laser therapy (HILT) is a novel laser treatment method derived from the basic principles of LLLT but operates at higher energy levels. HILT controls inflammation through anti-inflammatory and anti-edema effects, reducing prostaglandin, C-reactive protein, interleukin-1, and neopterin levels [13–15]. Its photomechanical effects provide rapid analgesia, enhance microcirculation, and contribute to local vasodilation, edema reduction, and metabolic support [16–19]. Furthermore, it inhibits nociception by activating A-fiber pathways and Melzack’s “gate control” mechanism, supported by the release of endogenous opioids [20, 21]. The photochemical effects of HILT promote ATP production by increasing mitochondrial RNA and DNA replication, thereby accelerating healing [22].

HILT differs from PBMT in its ability to deliver higher-powered beams (> 500 mW), enabling the transfer of substantial energy to deep tissues within a short period [23]. While HILT is widely used in the treatment of musculoskeletal disorders such as shoulder pain, lower back pain, and lateral epicondylitis, its effects on nerve regeneration remain inadequately studied. Given its higher energy delivery, HILT may support nerve regeneration more rapidly and effectively than PBMT. This study aims to evaluate the effects of HILT on sciatic nerve regeneration in rats following crush injury and compare its efficacy to PBMT.

## Materials and methods

### Animals and surgical procedure

All experimental procedures were performed in accordance with ethical guidelines and were approved by the ethical committee of the Animal Research Center at Bezmialem Vakif University (Istanbul,Turkey) (Project no. 2021/229) and supported by the Bezmialem Vakif University Research Fund (Project no. 20210208). In our study, sample size and power calculations were conducted based on 80% power (beta: 0.2) and 95% statistical significance (alpha: 0.05) using the G*Power program (Heinrich-Heine-Universität Düsseldorf, Düsseldorf, Germany). Thirty-three male Wistar-Albino rats, aged 8–12 weeks and weighing 220 ± 20 g, were obtained from the animal experimental center of Bezmialem Vakif University. Animals were housed in an animal facility under a 12-hour day/night cycle and were provided with food and water ad libitum. All animals underwent sciatic nerve crush injury and were randomly divided into three groups. The first group was left to heal spontaneously as the control group, the second group received HILT, and the third group received PBMT. Animals exhibiting signs of infection, abnormal weight loss, or death during or after the operation were excluded from the study.

### Surgical procedure

The animals received intraperitoneal injection of ketamine (60 mg/kg) and xylazine (6 mg/kg) combination for anaesthesia. Rats were immobilized on the operation table in a prone position then the left gluteal and lower extremity region of rats were shaved, and 10% povidone iodine solution was applied for antisepsis. A postero-lateral 2 cm incison to the gluteal tuberosity was made to pursue the hip joint lip on the right extremity and careful blunt dissections of the biceps femoris muscle was performed. The sciatic nerve was dissected from surrounding tissues from the sciatic incisura to the furcation area. A crush injury was applied to the left sciatic nerve of all animals using micro hemostatic forceps (Carl Martin, Solingen, Germany) for 30 s, ensuring the clamp encompassed the entire diameter of the nerve to induce axonotmesis according to the Seddon classification and a type 3 to type 4 injury according to the Sunderland classification. A 6 − 0 polipropilen suture (Katsan katgüt san. ve tic. aş, Izmir) was placed to the injury side for later identification. The deep muscle, subcutaneous tissue and skin were sutured with 4 − 0 resorbable PDS II (polydioxanone) suture (Johnson & Johnson, New Brunswick, NJ).

### Laser application protocol

The rats in the HILT group (n:11) were treated with a BTL-6000 High Intensity Laser 12 W (BLT Industries Ltd. 161 Cleveland way. Stevenage Hertfordshire. SGI 6BU. UK) that was applied gluteal surface directly above the site of injury, without making contact with the skin. The rats in the PBMT group (n:11) were treated with Low Level Laser (GRR trade.co.ltd. ,Ankara). PBMT was applied to the gluteal surface directly above the site of injury, making contact with the skin.The application parameters of both lasers were shown in Table 1. HILT and PBMT were started immediately after surgery and performed once every 3 days during the postoperative period. The tenth session was applied at the 27th postoperative day. All animals were sacrificed on postoperative day 30 using a high dose of anesthetic agents.

**Table 1:** Laser device parameters

Parameter	HILT	PBM Therapy
**Device information**		
Manufacturer	BTL	GRR LASER
Model identifier	BTL-6000 High Intensity Laser 12W	GRR LASER LTS04D
Emitter type	Class IV therapeutic laser	GaA1As
**Irradiation parameters**		
Beam shape	Divergent	N/A
Wavelength	1064 nm	650nm or 904nm
Operating mode	Continuous	Continuous
Frequency	N/A	0-100Hz
Peak radiant power	12W	80mW
Average radiant power	N/A	20mW
Beam shape	Circular	Circular
**Treatment parameters**		
Beam spot size	20mm	10mm
Applied power	2W	80mW
Exposure duration	60s	120s
Radiant exposure	60J/cm2	1.2J/cm^2^
Radiant energy	120J/session	2.4J/session
Number of points irradiated	One	One
Application technique	Non-contact mode	Contact mode
Number and frequency of treatment sessions	Once every 3 days for a period of 30 days 10 treatments total.	Once every 3 days for a period of 30 days 10 treatments total.
Total radiant energy	1200J	24J

### Sciatic functional index

The sciatic functional index analysis was used to evaluate the motor function of animals. SFI was evaluated pre-operatively and on 7th ,14th ,21st, 28th post-operative days. A walking hallway (8 × 11 × 70 cm), double-sided coated by carton box was prepared in advance. The ground of the walking alley was covered with white paper (10 × 60 cm). Rat’ s hindfoot were inked and rats were walked straight on the prepared walking hallway, printing their footprints on the paper. One acceptable footprints were selected for each rat and sciatic functional index parameters were measured. These parameters were distance between from the heel to the third toe (print length; PL), from first toe to fifth toe (toe spread; TS) and from second toe to fourth toe (intermediate toe spread; IT). The sciatic function index was calculated using the formula developed by Bain, Mackinnon and Hunter. For values obtained from 0 to −100, the value zero indicates the normal function, while the value − 100 indicates total loss of function.

### Electromyographic evalutaion

**Fig. 1:**
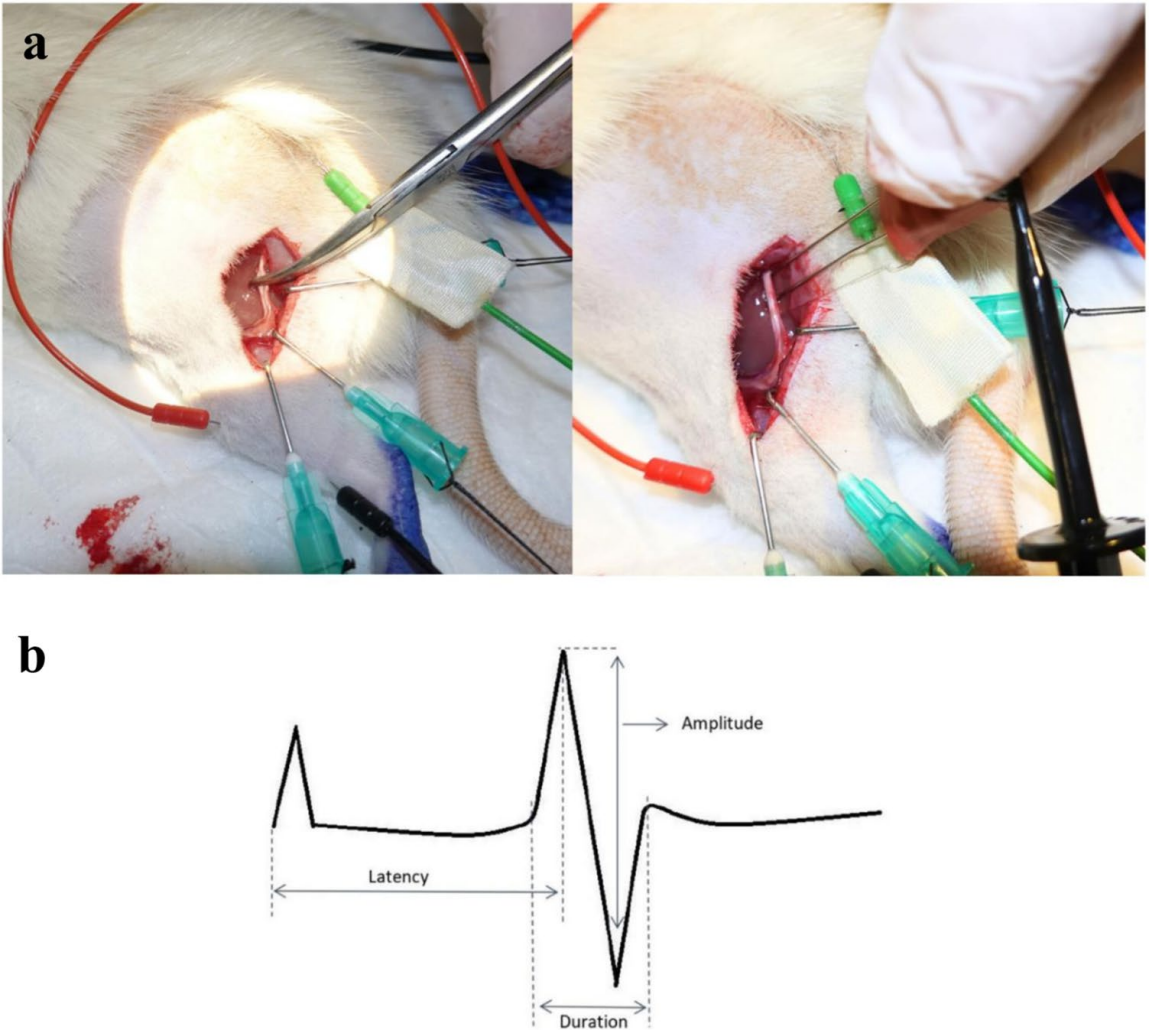
**EMG waves**

Electromyographic (EMG) measurements were conducted for all rats post-op (on days 30). EMG measurements were made with ADinstruments PowerLab 8/30 and ADinstruments Animal Bio amplifier devices. EMG Evaluations were made using the ADinstruments Lab Chart 7 program. (ADInstruments, Sydney, Australia) Two monopolar subdermal teflon needle electrodes (Spes Medica^®^, São Paulo, Brazil), each measuring 12 mm in length and 0.35 mm in diameter, were utilized for electrical impulses and recording. They were set up in parallel and spaced 5 mm apart. The stimulus electrodes were positioned 20 mm proximal from the trifurcation of the sciatic nerve and 5 mm proximal from the crush area of nerve. The active electrode was placed on the lateral head of the gastrocnemius muscle, while the reference electrode was positioned on the caudal part of the gastrocnemius muscle. The grounding electrode was placed between the stimulating electrode and the recording electrodes, directed cranially (Fig. 1.a). The electrical stimulation had a duration one times in five seconds total 5 times. Stimulation intensity was a 1 mA. The action potential of gastrocnemius was measured and three parameters were evaluated. Figure 1.b showed that the action potential was seen in the amplitude-time graph, negative peak and positive peak distance of compound muscle action potential implied amplitude(mV), The time between stimulus to initiate an evoked potential and a negative phase of action potential implied latency(ms). The time between start and end latency implied duration(ms). Three values were selected for each parameter, and the ratios of these values were statistically compared with each other.

### Histopathological and Histomorphometric Evalutaion

Nerve samples from the distal part of the injury were collected at the 30th postoperative day, and then, the animals were sacrificed. Harvested nerve tissues were fixed in 10% formaldehyde for 72 h and dehydrated in a series of graded alcohols Then, nerve samples were embedded in the paraffin blocks and were sectioned with a thickness of 5 μm using a microtome (RM2235, Leica, USA). The semi-thin sections were stained with toluidine blue (Sigma-Aldrich Co.) and examined using a photomicroscope (Carl Zeiss, Axio Observer, Germany) with 40x/0.95NA lens. The number of schwann cell per unit area and g- ratio were evaluated. the g-ratio was determined by dividing the inner axonal diameter by the outer fiber diameter and categorized into the ranges of 0–0.49, 0.50–0.54, 0.55–0.69, and 0.7–1. Values between 0.55 and 0.69 were regarded as optimum g-ratio [24].

### Statistical analysis

Repeated measurements of the Sciatic Functional Index score in the study were analyzed using a linear mixed model. Electromyography was analyzed in measurement results using the general linear recurrent measurement model. With this model, it was to assess the interaction between treatment and time, Bonferroni tests were utilized to compare groups in pairs. For the statistical evaluation of the number of Schwann cells, the Kruskal-Wallis test was used to find significant differences between groups for variances that were not normally distributed. Mann-Whitney U tests was used pairwise comparison of groups. The g-ratio values are presented as numbers (n) and percentages (p). The Fisher’s exact test and the Chi-squared test were both used to examine categorical data. The level *p* < 0.05 for all parameters was considered statistically significant. Statistical analyses of all data were carried out using SPSS software (Version 17, Chicago IL, USA).

## RESULTS

Autotomy were observed in four animals as postoperative complication. All animals responded favorably to laser exposure and survived until the end of the research except one animal in the control group.

### Walking track analysis and sciatic functional index

**Fig. 2:**
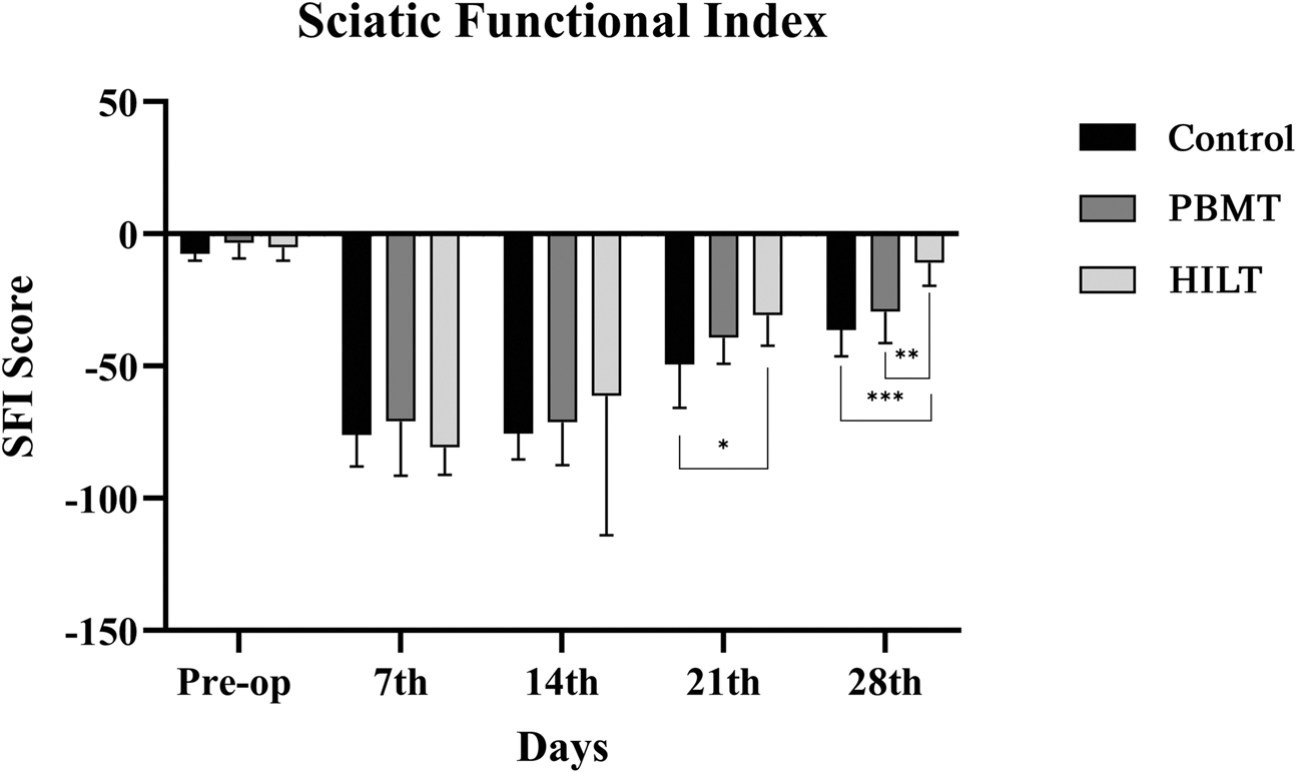
**Results of SFI distribution**. In the 21th week, a statistically significant was observed between HILT and control groups (*p* = 0.012). In the 28th week, the HILT group showed significantly lower SFI score than PBMT and control groups (*p* = 0.002, *p* < 0.001)

Autotomy was observed a total of four animals in the all experimental group*s.* Those animals were excluded from sciatic functional index evaluation. No significant differences were observed among the all groups at pre-op and post-op 7th and 14th days. In the 21st postoperative day, a statistically significant difference was observed between the HILT and control groups (*p* = 0.012; Fig. 2). In the 28st postoperative day, HILT group showed a statistically significant difference from the PBMT and control groups(*p* = 0.002, *p* < 0.001; Fig. 2). No significant differences were observed between PBMT and control groups (Fig. 2).

### Electromyographic evaluation

**Fig. 3:**
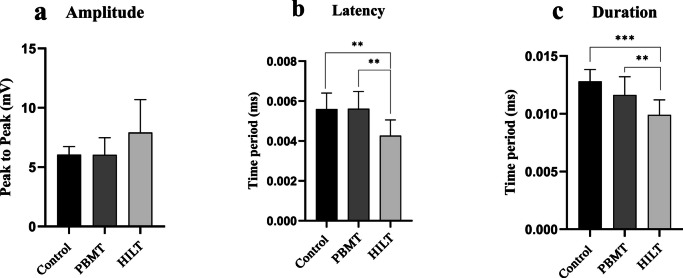
**Results of EMG distribution**. a No significant differences were observed among the all groups. b The HILT group showed significantly lower values compared to the PBMT (*p* = 0.002) and control (*p* = 0.003) groups. c The HILT group showed significantly lower values compared to the PBMT (*p* = 0.014) and control (*p* < 0.001) groups

Amplitude values in the HILT group (7.9 ± 2.7 mV) were higher compared to the PBMT (6.04 ± 1.43 mV) and control (6.05 ± 0.6 mV) groups (Table 2.) ; however, no significant differences were observed among the groups (Fig. 3a). The HILT group showed significantly better latency values compared to the PBMT (*p* = 0.002) and control (*p* = 0.003) groups, with no significant difference observed between the PBMT and control groups (Fig. 3b). The HILT group showed significantly better duration values compared to the PBMT (*p* = 0.014) and control (*p* < 0.001) groups, with no significant difference observed between the PBMT and control groups (Fig. 3c).

**Table 2:** Changes in Sciatic Funtional Index , amplitude, latency, duration (total and subscales) between the groups

	HILT group (n=11) mean (SD)	PBM group (n=11) mean (SD)	Control group (n=10) mean (SD)
Sciatic Functional Index (SFI)			
Before the damage	-5,11±4,97	-3,43±5,76	-7,61±2,53
Week 1 after injury	-80,79±10,3	-70,95±20,54	-76,01±11,84
Week 2 after injury	-61,25±52,6	-71,22±16,11	-75,64±9,56
Week 3 after injury	-30,72±11,52	-39,32±9,67	-49,47±16,34
Before sacrifice	-10,99±8,61	-29,37±11,96	-36,40±9,89
Amplitude(mV)			
Before injury	11,33±1,94	10,78±1,6	10,67±1,28
Before sacrifice	7,91±2,77	6,04±1,43	6,05±0,67
Latency(ms)			
Before injury	0,0036±0,0008	0,0037±0,0004	0,0034±0,0005
Before sacrifice	0,0042±0,0008	0,00561±0,0009	0,0056±0,0008
Duration(ms)			
Before injury	0,0071±0,0013	0,0073±0,0005	0,007±0,001
Before sacrifice	0,009±0,0013	0,011±0,0015	0,013±0,001

### Histopathological findings

**Fig. 4:**
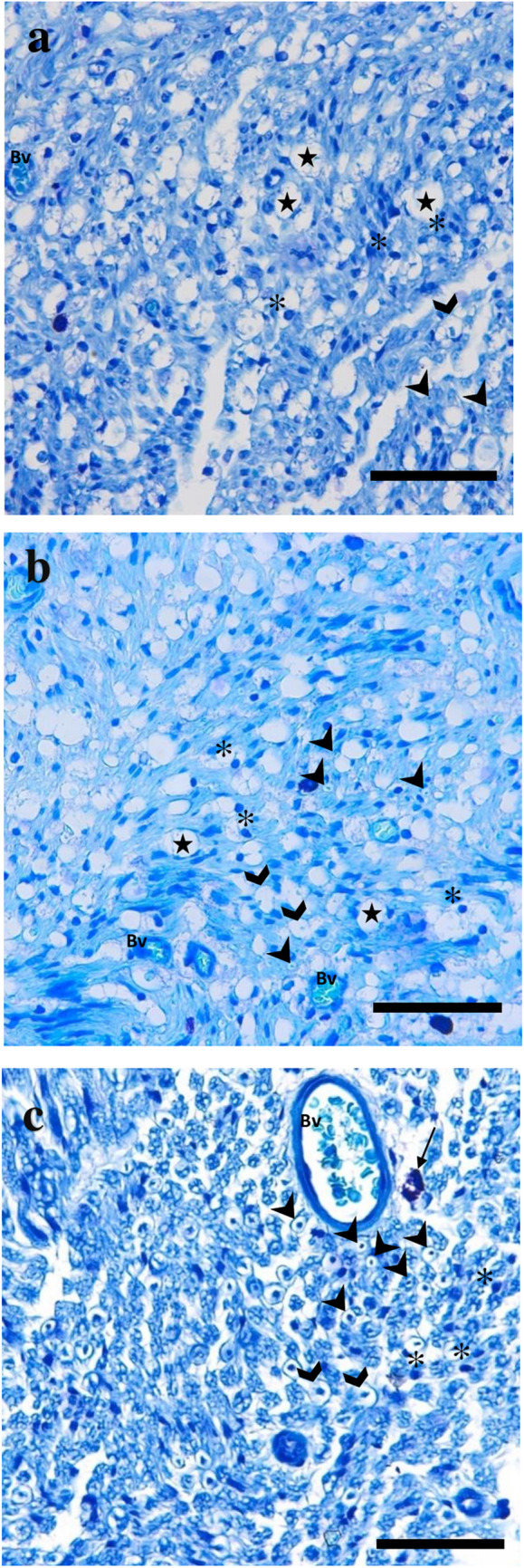
**Representative images of thin sections of the nerves**. a The control group demonstrate multiple Schwann cells, severe degenerative changes in the myelin sheaths of axons. b The PBMT group demonstrating accumulation of thin membrane-bounded vacuoles and higher number of the Schwann cells. c HILT group demonstrating higher number of the myelinated axons and physiological structure is well. Bars in semi-thin sections = 13 μm. ➤: myelinated nerve axon, ✱: Schwann cell, ★: vacuole, ❱: degenerative myelinated axon, ➞: macrophage, **Bv**: capillary blood vessel. Toluidine blue (T3260, Sigma-Aldrich), light microscope (Carl Zeiss, Axio Observer) with 40x/0.95NA objective

In all groups, sections from the proximal side of the injury area was histopathologically examined. Myelin sheath degeneration in large-diameter axons was lower in the treated groups compared to the control group. During the examination of tissue sections in the PBMT group, degenerative myelinated axons were observed, similar to those in the control group (Fig. 4a and b). Moderate-intensity myelinated axons, a large number of Schwann cells, fibrotic tissue and blood vessels were clearly observed (Fig. 4a**)**. Examination of tissue sections from the HILT group revealed a normal overall nerve structure. A relatively higher number of myelinated axons were observed compared to the other groups. In addition, Schwann cell number and fibrotic tissues were relatively lower (Fig. 4c).

### Histomorphometric findings

**Fig. 5:**
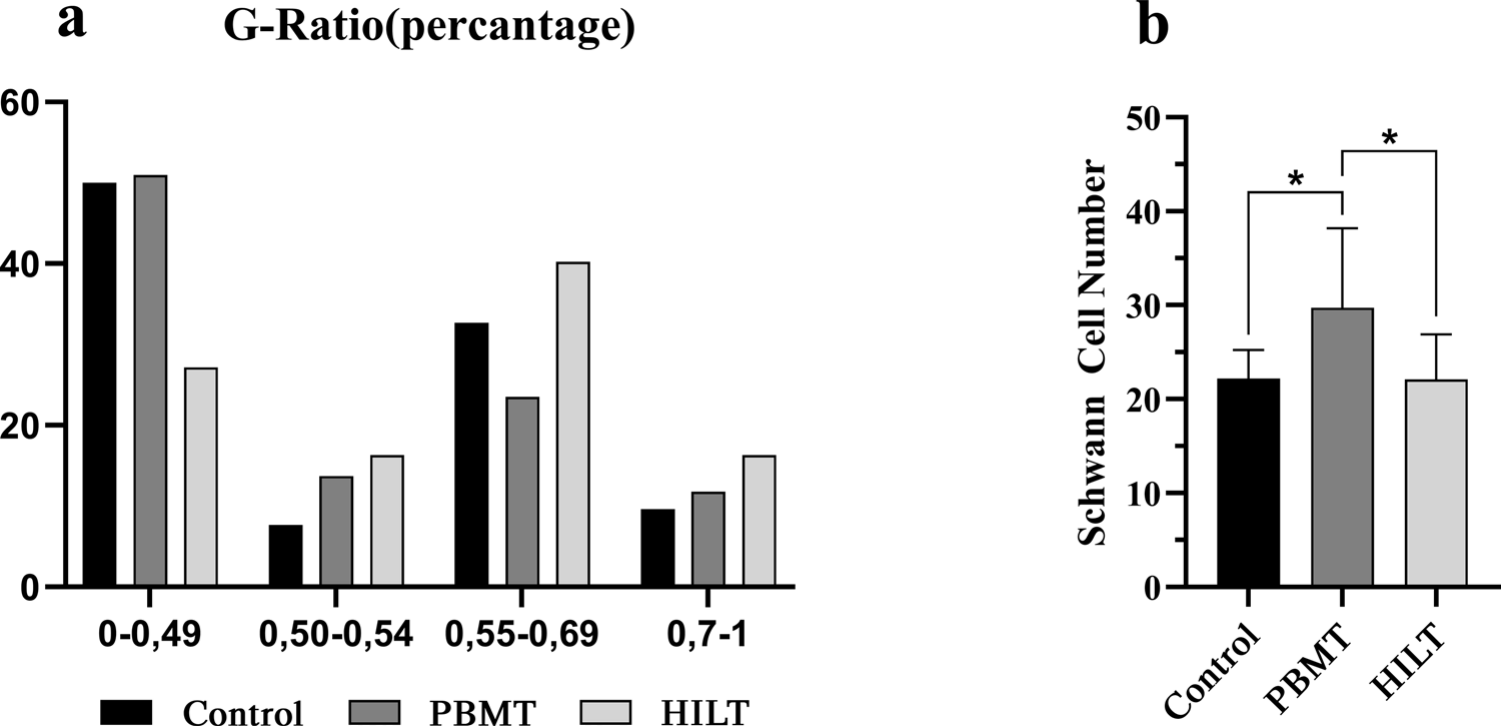
**Results of Histopathological analysis** a Analysis of the g-ratio results revealed no significant differences among groups. The HILT presented better results at optimal g-ratio range (0.55–0.69). b The number of Schwann cell per unit square micrometer was significantly higher in the PBMT group when compared with the control group (*p* = 0.0370) and HILT group (*p* = 0.048)

All Schwann cells were counted in the cross section of the nerve samples divided by total area and the number of Schwann in the per unit area was calculated. Schwann cell density in the PBMT group was significantly higher than HILT (*p* = 0.048 and control (*p* = 0.0370) groups. No significant differences were observed between HILT and control groups (Fig. 5b). G-ratio values in the HILT group presented better results, since most axon fibers found to be in optimal g-ratio range (0.55–0.69). Insufficient myelination was observed in the PBMT group, which had a higher value of axons concentrated in the g-ratio range of 0–0.49.However, in the HILT group, the number of nerve fibers with suboptimal g-ratio ranges of 0–0.49 is lower than other groups, which indicates a better rate of myelinization in the HILT group (Fig. 5a).

## DISCUSSION

Laser therapy is a non-invasive method that supports tissue regeneration and has a wide range of applications [25]. Photobiomodulation therapy (PBMT) is particularly effective in musculoskeletal disorders and nerve injuries. Studies in the literature have demonstrated that the biostimulatory effects of PBMT increase myelin sheath thickness, promote Schwann cell proliferation, and exert positive effects on neurotrophic growth factors [9–11].

High-intensity laser therapy (HILT) has emerged as an alternative method to PBMT by delivering stronger energy applications. The advantages of HILT include deeper tissue penetration, shorter treatment duration, and higher energy doses. These features facilitate tissue regeneration, improve local blood circulation, and enhance thermal and mechanical effects [15, 26].

Moreover, HILT induces electromagnetic fields, photoelectric changes, and electrochemical reactions in target tissues, contributing to pain and edema reduction [20, 21]. This study aims to investigate the effects of HILT on sciatic nerve regeneration following crush injury and to fill this critical gap in the literature.

Literature reviews indicate successful outcomes in studies involving low-level lasers. Khullar et al. [27] demonstrated the effects of low-level laser therapy (GaAlAs 830 nm, 6 J) on nerve regeneration following axonotmesis, with daily applications over 28 days. The PBMT group exhibited improved functional recovery of the nerve. Similarly, Gomes et al. [28] used a low-intensity laser with a wavelength of 660 nm and a dose of 3.84 J/cm², applied for 5 min daily over 21 days. Histomorphometric evaluations revealed that tissue regeneration in the laser-treated group was both qualitatively and quantitatively superior to the placebo group.

The aim of our experimental study was to evaluate the effects of HILT (High-Intensity Laser Therapy) on sciatic nerve regeneration in rats following crush injury and to compare its efficacy with PBMT (Photobiomodulation Therapy).

On postoperative days 14 and 21, the Sciatic Functional Index (SFI) demonstrated better recovery in the HILT group compared to the PBMT group (Table 2 ; Fig. 2). Electromyography (EMG) was analyzed to confirm the reinnervation of distal motor units. The data revealed no statistically significant differences in amplitude values among the HILT, PBMT, and control groups. However, latency and duration parameters were significantly higher in the PBMT group compared to the control and HILT groups (Table 2 ; Fig. 3) .

In the HILT group, the percentage of nerve fibers within the optimal g-ratio range (0.55–0.69) was higher, while the percentage in the suboptimal range (0–0.49) was lower compared to the other groups, indicating better myelination. The PBMT group exhibited a relatively higher number of Schwann cells, which was statistically different from the other groups. This suggests that the excessive number of Schwann cells in the PBMT group reflects ongoing degeneration, while the lower axon count within the optimal g-ratio range indicates delayed regeneration. In contrast, the HILT group demonstrated a lower number of Schwann cells and a higher axon count within the optimal g-ratio range, suggesting an accelerated regeneration process (Fig. 5). Consistent with findings from the Sciatic Functional Index, electromyography, and histopathological examinations, it can be hypothesized that HILT treatment may enhance nerve regeneration by delivering a high amount of energy to deep tissues in a short period through stronger laser beams.

The effects of high-intensity laser therapy (HILT) may stem from its ability to regulate the cytoskeletal network, contributing to tissue regeneration and repair. This therapy positively influences endothelial cell functions by increasing extracellular matrix and fibronectin production by connective tissue cells [21]. The limited number of studies using HILT has resulted in a restricted understanding of the treatment. Some preliminary research has shown that HILT is more effective than LLLT due to its higher intensity and the ability of the laser to penetrate deeper. The findings from our study on nerve regeneration support previous studies conducted in other fields.

The literature contains numerous studies examining the wavelength, dosage, application duration, and session intervals in laser treatments. However, in studies focusing solely on laser parameters, it remains unclear whether the primary influencing factor is wavelength or energy. The effectiveness of the treatment also depends on other factors, such as power, dosage, and laser type. Additionally, the expected regeneration time varies based on the type of injury, the nerve being studied, and the species examined [29], making it challenging to compare treatment protocols across studies.

The regenerative effects of different wavelengths in low-intensity laser therapy have been investigated in several studies. Barbosa et al. [30] compared the effects of low-power GaAlAs lasers (660 nm and 830 nm) on sciatic nerve regeneration following crush injuries, applied for 21 consecutive days. On the 14th postoperative day, the 660 nm group showed significant differences compared to the sham and 880 nm groups. They concluded that the 660 nm GaAlAs laser promoted early functional nerve recovery compared to the other groups. In contrast, Diker et al. [24] examined the therapeutic effects of 660 nm and 880 nm GaAlAs lasers (2.7 J/session) on inferior alveolar nerve (IAN) regeneration following crush injury. Laser therapy was initiated immediately after surgery and applied every 3 days for 30 days. Their findings indicated that the 880 nm wavelength, with its higher tissue penetration capacity, was more effective for biostimulation of the IAN after injury. These studies highlight contradictory findings regarding the optimal wavelength for nerve regeneration. The variations in results are attributed to differences in laser energy, techniques, and application durations.

Additionally, studies have investigated the tissue penetration capabilities of lasers with different wavelengths. Takhtfooladi and Sharifi examined the effects of 680 nm PBMT, 650 nm red LED, and 450 nm blue LED treatments on nerve regeneration after end-to-end suturing of the severed sciatic nerve over 14 days. They reported a higher number of total neurons, myelinated axons, and Schwann cells in the low-intensity laser group compared to the other groups [31]. The study concluded that a high-intensity laser with a wavelength of 1064 nm was more effective than a low-level laser with a wavelength of 650 nm. This finding suggests that lasers with longer wavelengths, offering higher tissue penetration capacity, may be more advantageous for biostimulation following nerve injury.

In the literature, it has been reported that the amount of energy applied in laser biostimulation treatments may influence healing effectiveness. Marcolino et al. [32] compared the regenerative effects of 10 J/cm², 40 J/cm², and 80 J/cm² of AsGaAl laser (830 nm) over 21 consecutive days following a sciatic nerve crush injury. They observed enhanced functional recovery on the 7th postoperative day in the 40 J/cm² laser irradiation group compared to the sham group. Additionally, on the 14th postoperative day, both the 40 J/cm² and 80 J/cm² laser irradiation groups demonstrated better outcomes compared to the sham group, while no differences were noted among the sham, 10 J/cm², 40 J/cm², and 80 J/cm² groups on the 21st postoperative day. The study concluded that PBMT at fluencies of 40 J/cm² and 80 J/cm² had a positive effect on accelerating functional nerve healing. In the present study, HILT was administered with a radiant energy of 120 J per session, while PBMT was administered with a radiant energy of 2.4 J per session. However, our results showed that HILT was more effective than PBMT.

There are numerous studies on the application of laser treatments. However, there is no established protocol. One study reported that 20 sessions over a total period of 3 months would be sufficient for treating IAN injury in humans [33]. In many nerve regeneration studies on rats following axonotmesis injury, it has been noted that functional recovery occurs between 4 and 8 weeks [34]. Therefore, in this study, to observe the bioconcentration and regenerative effects on crush-type injury in the sciatic nerve, irradiation with HILT and PBMT was administered 3 days a week for a total of 10 sessions.

In the literature comparing HILT and LLLT, Alayat et al. [35] investigated and compared the effects of high-intensity laser therapy (HILT) and low-level laser therapy (LLLT) in the treatment of patients with Bell’s palsy. The results indicated that both HILT and LLLT are effective physical therapy modalities for the recovery of Bell’s palsy patients, with HILT showing a slightly greater improvement than LLLT. Similarly, Kheshi et al. [36] compared the effects of LLLT and HILT on pain relief and functional improvement in patients with knee osteoarthritis. Their findings revealed that HILT combined with exercises was more effective than LLLT combined with exercises, and both treatments were superior to exercise alone for managing knee osteoarthritis.

A limitation of this study is the lack of evaluation of the heat induced during PBMT (Photobiomodulation Therapy) application. Accurately modeling temperature changes in in vivo studies is challenging. In the literature, Capon et al. [37] have reported that lasers transmit energy to surrounding tissues in the form of heat, creating a temperature gradient of 45–50 °C in adjacent tissues, regardless of the type of laser used. During the wound healing process, this temperature increase is suggested to result from the response of viable cells and to induce a heat shock response (HSR) by causing temporary changes in cellular metabolism due to the supra-physiological heat. This phenomenon is associated with the production of proteins known as HSPs (Heat Shock Proteins). However, the study by Wang et al. [38] demonstrated that the heat generated by laser-tissue interaction does not lead to significant changes in hemodynamic and metabolic effects. Additionally, the generalizability of the crush injury model used in this study is limited. Further controlled clinical studies are needed to investigate the effects of different types of nerve injuries, the underlying mechanisms of regeneration, and the impact of laser parameters. Finally, many previous studies have not adequately defined critical parameters of laser irradiation, such as dose, power, irradiation duration, and application method. This lack of standardization complicates comparisons between studies and contributes to inconsistent findings.

## Conclusion

Based on the sciatic functional index (SFI), electrophysiological evaluations, and histomorphometric results of the present study, it can be concluded that after a 30-day healing period, HILT had a more pronounced positive effect on nerve regeneration compared to PBMT and the control groups. Although PBMT also contributed to functional recovery, the difference was not statistically significant. Due to its technical advantages, such as greater tissue penetration, shorter therapy duration, and higher energy doses, HILT is preferable for nerve biostimulation following injury. Consequently, HILT emerges as a promising new technology for treating nerve injuries in clinical settings. Further research is necessary to determine the optimal HILT settings for effective biostimulation of injured nerves.
